# Accessing HIV care services by key populations — An Ubuntu philosophy reflection

**DOI:** 10.4102/curationis.v48i1.2633

**Published:** 2025-04-30

**Authors:** Idah Moyo, Livhuwani Tshivhase

**Affiliations:** 1 Department of Health Studies, College of Human Sciences, University of South Africa, Pretoria, South Africa

**Keywords:** access, Africa, HIV care services, key populations, Ubuntu

## Abstract

**Background:**

Key populations are disproportionately affected by HIV despite the significant decrease in new HIV infections in Africa. They experience challenges like stigma and discrimination as they interface with the healthcare system. This results in reduced access to HIV care services for key populations. Therefore, the attainment of HIV epidemic control may not be easily realised if these gaps are not addressed.

**Objectives:**

To explore and synthesise factors associated with accessing HIV care services by key populations, as well as make a reflection of this process using Ubuntu philosophy.

**Method:**

An integrative literature review was conducted on studies published between 2014 and 2024. An electronic search was performed on several databases. Examples of key phrases that were utilised for the search included Africa, HIV care services, key populations and Ubuntu. The studies included were qualitative and quantitative from peer-reviewed journals and restricted to Africa.

**Results:**

The following themes emerged: non-inclusive healthcare environment, attitudes of healthcare workers and stigma and discrimination. These findings illustrate the challenges and barriers affecting access to HIV care services for key populations.

**Conclusion:**

The insights from this review call for a paradigm shift in the training programmes of healthcare providers in Southern Africa.

**Contribution:**

Given the challenges that affect key populations as they access HIV care services, in-service and pre-service training of healthcare providers should incorporate the humane values of Ubuntu.

## Introduction

The prevalence of HIV among key populations (KP) is disproportionately higher than in the general population. This poses a challenge towards the realisation of the UNAIDS targeted goals of the 95-95-95: that is 95% of the HIV infected know their status, 95% of those diagnosed as positive to be on treatment and 95% of those on treatment to be virally suppressed by 2025 (Rasweswe et al. 2023). According to the Joint United Nations Programme on HIV/AIDS (UNAIDS) (2023), since 2010, the world has had a significant decrease in new HIV infections with eastern and southern Africa recording a 57% decrease in 2022. Despite the observed decrease in new HIV infections, gaps still exist with key population groups disproportionately affected by the HIV epidemic. According to the UNAIDS (2023), the HIV prevalence among key populations compared with adults (aged 15–49 years), in eastern and southern Africa for the period 2022, was disaggregated as follows: 29.9% among female sex workers (FSW); 12.9% among gay men and other men who have sex with men (MSM); 21.8% among people who inject drugs and 42.8% among transgender people. This is compared to an HIV prevalence of 5.9% for adults (15–49 years) in the general population (UNAIDS 2023).

In an Indonesian study by Fauk et al. (2021), some nurses and doctors confessed to personally discriminating and stigmatising HIV-positive patients. In a related study, Wouters et al. (2022) found evidence that stigma and discrimination against people living with HIV, impacted negatively on the uptake of HIV care services. On the other hand, Chimoyi et al. (2021) revealed that stigma and discrimination against the HIV-positive resulted in a restricted uptake and adherence to treatment and care. A study in Southern Africa by Müller et al. (2018) established that sexual and gender minority adolescents experienced negative attitudes from healthcare providers. The United Nations Population Fund (2017) defines the sexual and gender minority adolescents as those identifying as lesbian, gay or bisexual (sexual minority adolescents) and adolescents identifying as transgender or gender non-conforming, as gender minority adolescents. Müller et al. (2018) further suggested that these adolescents experienced reduced access to sexual and reproductive healthcare services. Kigombola et al. (2023) found that key populations experienced judgemental attitudes and stigma from healthcare workers as they accessed healthcare services. As nurses are the backbone of the healthcare system (Salmond & Macdonald 2021), it is therefore prudent to address stigma and discrimination by creating an awareness on Ubuntu philosophy through pre-service and in-service training. The paradigm shifts in the mindset of healthcare personnel postulated by Wodajo, Thupayagale-Tshweneagae and Akpor (2017) can be achieved by using local contexts such as Ubuntu, incorporating societal norms and human rights.

There is evidence that key populations suffer from stigma and discrimination from healthcare providers. Studies by Duby et al. (2019) in South Africa, Hunt et al. (2017) in Zimbabwe and Graham et al. (2018) in Kenya attest to that. Stigma and discrimination make it difficult to access HIV care services. This is further compounded by the negative attitudes of nurses towards key populations (Matovu et al. 2019). According to Rasweswe et al. (2023) if nurses received a sensitisation training informed by Ubuntu philosophy, there would be positive health outcomes. This article argues that the same would be realised with regard to key populations. Mokhachane et al. (2023) define Ubuntu as an embodiment of a humanistic ideal and a foundational principle of professionalism from a Southern African perspective. Magezi and Khlopa (2021) ably argue and agree that Ubuntu is not just a descriptive and regulatory view, but encompasses norms and ethics of African society about how people should relate to each other. This is important with regard to how healthcare workers treat key populations as Ubuntu considers individuals as whole persons with innate dignity that cannot be defiled. This resonates with Virginia Henderson’s holistic view of nursing (Henderson 1969).

In fact, Ewuoso (2021:34) implies that with regard to confidentiality, there are similarities between Ubuntu philosophy and aspects of the Hippocratic Oath and the nurses’ pledge, which medical practitioners and nurses take. Contrary to the provisions of the Hippocratic Oath and Ubuntu, there have been reports of perceived breaches of confidentiality in HIV care settings (Bayisa et al. 2022) in Ethiopia and Malawi (Olawepo et al. 2021). Ewuoso et al. (2021) argue that whilst the Judeo-Christian Islam approach to human rights attaches spirituality to the rights a person has, Ubuntuism holds that human dignity is inherent in an individual as a physical being. This has implication on how healthcare providers treat patients regardless of their sexual orientation.

The Ubuntu philosophy of compassion resonates with the nursing ethical code as stated by Florence Nightingale, but it goes further to express solidarity ‘Umuntu ngumuntu ngabantu – I am because you are’. If healthcare providers adopted this approach, there would be better health outcomes (Mokhachane et al. 2023). This article is an integrative review that explores the barriers encountered by key populations as they access HIV prevention, treatment and care services. It further contends that these challenges could be further addressed by sensitisation informed by Ubuntu philosophy of healthcare workers.

### Aims of the review

The aim of this integrative literature review was to explore and synthesise factors associated with accessing HIV care services by key populations and make a reflection using the Ubuntu Philosophy.

## Methods

An integrative literature review was performed. Integrative reviews are considered an appropriate approach to gain an in-depth understanding of the phenomenon under study and facilitate generalisation of study results as articulated by Whittemore (2005). In addition, Whittemore and Knafl (2005) posit that integrative reviews combine qualitative and quantitative studies and allow for the inclusion of diverse methodologies, in contrast to systematic reviews and meta-analyses which only include quantitative studies of similar methodology.

### Search methods

A comprehensive electronic search was conducted on the following databases: Medline; PubMed; EBSCOhost: Africa-wide information, Science Direct, Health Source: Google Scholar and Academia Edition for articles on key populations and their access to HIV care services published between 2010 and 2024. The search terms that were utilised by the researchers included key populations, HIV care services, access, challenges and Ubuntu philosophy.

### Inclusion and exclusion criteria

This integrative review included primary studies that were published in English. The inclusion criteria encompassed studies published in peer-reviewed journals for the period between 2014 and 2024. The researchers anticipated that the selected period would provide relevant articles and adequate recent evidence related to the topic under study. Additionally, the authors assumed that in the sub-Saharan countries with a high HIV burden among key populations and a reduced access to healthcare services, efforts have been made to improve access and service quality. In addition, a few studies related to key populations and their access to HIV care services have been done mostly in the past 10 years. The review included both qualitative and quantitative studies. Articles were also excluded if their study settings were not healthcare ones because the focus of the review was on factors that would affect HIV care service access for key populations. In addition, the studies were not included if they had not been conducted in the African region.

### Search results

**FIGURE 1:**
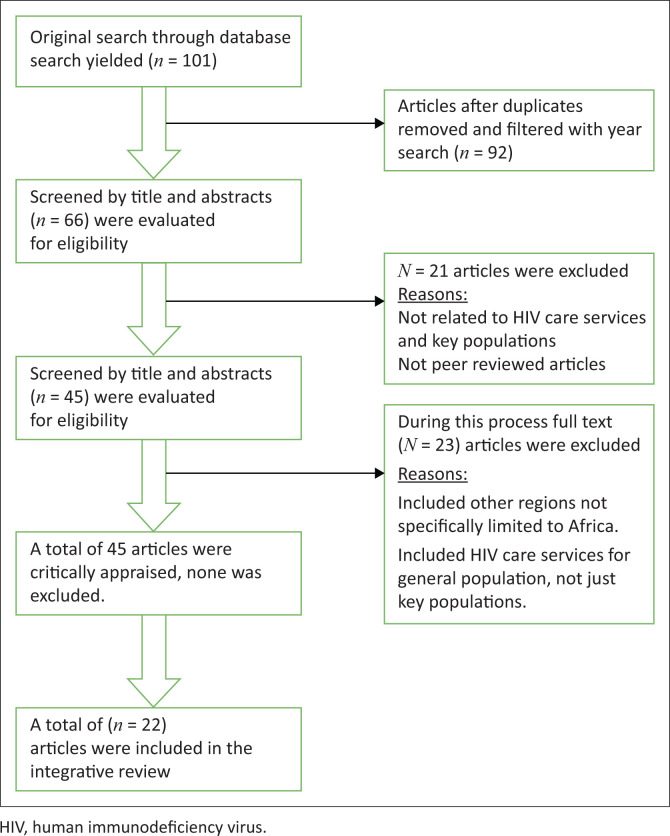
Flowchart on selection process and final number of articles selected.

Our search for published articles yielded 101 records that were initially reviewed. This was followed by further screening and examination of the titles and abstracts of 66 records. This process included the removal of duplicates and 21 records were excluded. A total of 45 records were further evaluated for eligibility, with 23 records removed as they did not meet the inclusion criteria. Finally, a total of 22 studies were included in this integrative review as depicted in Figure 1.

### Data analysis

Qualitative content analysis was utilised to analyse the data as described in some studies (Hsieh & Shannon 2005; Lopes & Decesaro 2014; Sandelowski, Voils & Barroso 2006). The findings from all the articles that were included, were read several times and coded when patterns were identified. According to Weber (1990), the basic coding process in content analysis involves organising large quantities of text into smaller content categories. These categories or patterns or themes emerged from the analysis. A summary of all the studies included in this review is shown in Table 1. The qualitative appraisal checklist is presented in Table 2, whilst the quantitative appraisal checklist is in Table 3.

**TABLE 1:** Summary of all articles included in the literature review.

Author	Setting & sample	Design	Findings
Kushwaha et al. (2017)	Ghana	Interpretive description qualitative study	Negative healthcare climates.
Ross et al. (2014)	Tanzania MSM	Quantitative	Unwelcoming healthcare environment restricting access to HIV care services.
Wanyenze et al. (2016)	Uganda MSM	Qualitative research design	Environment not conducive, MSM were not comfortable to disclose their sexual orientation to providers of care.
Ssekamatte et al. (2020)	Uganda Trans-women sex workers	Exploratory qualitative study	A non-inclusive health environment was characterised by healthcare system barriers: social exclusion, stigmatisation and breaches of confidentiality and non-flexible clinic hours of operation for key populations.
Nyato et al. (2019)	Tanzania FSW	Qualitative study	Absence of a conducive environment for accessing prevention, care and treatment services was noted.
Nnko et al. (2019)	Sub-Saharan Africa	Qualitative	A conducive healthcare environment was associated with client satisfaction, improved privacy, timely services, good reception and non-discriminatory attitudes of healthcare providers.
Kigombola et al. (2023)	Tanzania Key populations (MSM & FSW)	Qualitative research design	Lack of KP-friendly services affected the utilisation of services.
Kushwaha et al. (2017)	Ghana MSM and healthcare provider	Qualitative research design	Negative healthcare climates. Lack of trust in the healthcare system by recipients of care.
Kapanda et al. (2019)	Malawi health professions cadres	Qualitative study	Positive attitude is displayed by healthcare providers towards providing HIV care services for key populations.
Scheibe et al. (2017)	Kenya	Mixed-methods approach	In-service training of healthcare providers on understanding and counselling of the MSM as they accessed HIV care services resulted in improved client care. Following the training, there was a reduction in negative attitudes towards the MSM, and nurses were able to provide non-discriminatory care.
Richter and Buthelezi (2021)	Africa	Qualitative	Positive experiences of sex workers at the few sex work-specific clinics. Perceived negative attitudes of healthcare workers on sexual practices of sex workers.
Fobosi et al. (2017)	South Africa Sex workers at truck-stop clinics	Mixed-methods approach	Satisfaction with friendly healthcare providers. Provision of differentiated and flexible healthcare services (providing services at night).
Mulemfo, Moyo and Mavhandu-Mudzusi (2023)	South Africa LGBTQI+	Interpretive phenomenological Analysis design	Heterocentric healthcare service approaches were found to exclude LGBTQI+ people from accessing HIV care services.
Spyrelis and Ibisomi (2022)	Southern Africa	Exploratory rapid assessment study	Sex workers reported experiencing stigma and discrimination when accessing healthcare services.
Mokhwelepa, Ngwenya and Sumbane (2024)	South Africa Sex workers	Qualitative	Access to healthcare services by sex workers is affected by stigma and discrimination prevailing in healthcare facilities.
Krishnaratne et al. (2020)	Zambia and South Africa FSW & MSM	Quantitative	High levels of stigma and judgement by healthcare workers towards key populations, affecting access to healthcare services.
Duby et al. (2019)	South Africa Healthcare providers, MSM & FSW	Mixed method	Training of healthcare providers in key population friendliness, resulted in improved attitudes, increased empathy for key populations, and a reduction in negative and discriminatory & judgemental attitudes.
Moyo and Macherera (2021)	Zimbabwe FSW	Descriptive phenomenological study	Stigmatisation of affected interfered with retention in care and recipients of care self-transferred to other facilities, pushing some study participants to self-transfer.
Graham et al. (2018)	Kenya gay, bisexual, and other MSM	Qualitative	Stigma and discrimination were prevalent across healthcare facilities and this contributed to mental health or substance abuse problems.
Hunt et al. (2017)	Zimbabwe	Qualitative	Stigma and discrimination in healthcare facilities were found to be a key barrier for key populations to access HIV care services. The key populations were not to discuss their sexual orientation with healthcare workers and experienced psychological distress.
Spyrelis, and Ibisomi (2022)	Southern African Development Community (SADC) countries namely: Lesotho; Malawi; South Africa; Eswatini; and Zambia	Qualitative	Some sex workers complained about nurses being rude to them, insulting them during clinic visits and blaming them for contracting illnesses, especially STIs.
Shannon et al. (2018)	Global	Quantitative	Despite the high burden of HIV pandemic amongst sex workers, there is still suboptimal coverage and retention of prevention and treatment, alongside structural barriers of criminalisation, stigma and discrimination.

Please see the full reference list of this article, Moyo, I. & Tshivhase, L., 2025, ‘Accessing HIV care services by key populations — An Ubuntu philosophy reflection’, Curationis 48(1), a2633. https://doi.org/10.4102/curationis.v48i1.2633 for more information.

MSM, men who have sex with men; FSW, female sex workers; KP, key populations; HIV, human immunodeficiency virus; STIs, sexually transmitted infections; LGBTQI+, Lesbian, Gay, Bisexual, Transgender, Queer, Intersex and other.

**TABLE 2:** Qualitative studies critical appraisal checklist.

Number	Criteria	Yes	No
1	Congruity between stated philosophical perspective and research methodology	15	1
2	Congruity between methodology and research question or objective	16	0
3	Congruity between methodology and methods used to collect data	16	0
4	Congruity between methodology and representation and analysis of data	16	0
5	Congruity between methodology and interpretation of results	16	0
6	There is a statement locating the researcher culturally or theoretically	0	16
7	The influence of the researcher on the research and vice versa is addressed	16	0
8	Participants and other voices are adequately represented	16	0
9	Ethical according to current criteria, evidence of ethical approval	16	0
10	Conclusions drawn flow from analysis or interpretation of data	16	0

*Source*: Pearson, A., 2004, ‘Balancing the evidence: Incorporating the synthesis of qualitative data into systematic reviews’, *JBI Reports* 2(2), 45–64. https://doi.org/10.1111/j.14796988.2004.00008.x

**TABLE 3:** Quantitative studies critical appraisal checklist.

Number	Criteria	Yes	No
1	Aims and objectives clearly stated	6	0
2	Hypothesis or research question clearly specified	6	0
3	Dependent and independent variables clearly stated	6	0
4	Variables adequately operationalised	6	0
5	Design adequately described	6	0
6	Method appropriate	6	0
7	Instrument used tested for reliability and validity	6	0
8	Sample, inclusion or exclusion and response rate described	6	0
9	Statistical errors discussed	6	0
10	Ethical consideration	6	0
11	Was the study piloted?	5	1
12	Statistical analysis appropriate	6	0
13	Results reported and clear	6	0
14	Results reported related to hypothesis and literature	5	1
15	Limitations reported	6	0
16	Conclusions do not go beyond limit of data and results	6	0
17	Findings able to be generalised	6	0
18	Implications discussed	6	0
19	Conflict of interest with sponsor	0	6
20	Data available for scrutiny and re-analysis	3	3

*Source*: Bowling, A., 2009, *Research methods in health: Investigating health and health services*, Open University Press, Maidenhead

### Ethical considerations

This article followed all ethical standards for research without direct contact with human or animal subjects.

## Results

### Study demographics

This review was restricted to studies conducted in Africa. A total of 22 articles that met the inclusion criteria were included in the study. The researchers deliberately chose to restrict the study to articles from African countries, assumed to be having similar or related characteristics, including the way key populations are viewed. Most of the articles that met the inclusion criteria were qualitative in nature (*n* = 16), whilst of the remaining six, the quantitative were (*n* = 3) whilst those with mixed methods constituted (*n* = 3). The 22 studies that were included in this review were disaggregated as follows: 50%, 30% and 20% from southern, eastern and western Africa, respectively.

### Study findings

**FIGURE 2:**
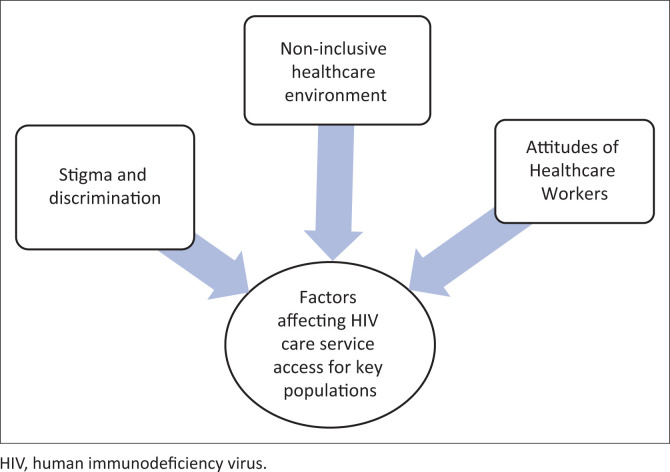
Summary of themes.

An analysis of all reviewed articles yielded the following themes: non-inclusive healthcare environment, attitudes of healthcare workers and stigma and discrimination as displayed in Figure 2. On the other hand, Magezi and Khlopa (2021) argue that an Ubuntu environment is one characterised by care, compassion and empathy for someone in distressed circumstances. The discussion demonstrates how the absence of Ubuntu yields a negative and non-conducive healthcare environment.

### Non-inclusive healthcare environment

A study in Ghana by Kushwaha et al. (2017) found that the MSM did not trust the healthcare system as they felt that it was unfriendly and unsupportive of their basic human rights to respect, equitable treatment and free expression. With reference to the MSM community and their access to HIV care services in Tanzania, prior studies have established that an unwelcoming healthcare environment was the contributory factor to reduced access to HIV services (Ross et al. 2014). According to Wanyenze et al. (2016) in a study in Uganda, the MSM were not comfortable to disclose their sexual orientation to providers of care and also felt that the healthcare workers did not respect them. Studies by Ssekamatte et al. (2020) in Uganda and Nyato et al. (2019) in Tanzania that involved trans-women and sex workers found the healthcare environment non-inclusive. Evidence from these studies suggested that the operating hours were inflexible, the environment characterised by stigma and breaches of confidentiality. In contrast, a systematic review conducted in Southern Africa by Nnko et al. (2019), noted that a conducive healthcare environment was characterised by improved privacy, client satisfaction, timely services, good reception and non-discriminatory attitudes of healthcare providers amongst other things.

### Attitudes of healthcare workers

Kigombola et al. (2023) in a study in Tanzania found that the utilisation of healthcare services was affected by the fact that the providers of care were not trained in the provision of key population-friendly HIV care services. In turn, the recipients of care reported that they encountered judgemental attitudes and stigma from healthcare workers during the clinic visits. A study in Uganda found that there was a cocktail of barriers to access to HIV treatment amongst FSW and MSM. These included negative attitudes of healthcare workers and lack of skills set in managing key populations (Ssekamatte et al. 2020). Similarly, the negative attitudes of healthcare workers towards the MSM were also noted in a study in Ghana (Kushwaha et al. 2017). A South African study by Mulemfo et al. (2023) found that the healthcare environment was unfriendly to key populations, making access to HIV care services difficult. However, positive attitudes were noted amongst providers of care towards serving key populations as the following studies demonstrate. According to Kapanda et al. (2019) in a study in Malawi, healthcare providers had a positive attitude towards providing HIV care services for key populations. The same healthcare providers felt that they had an obligation to meet the unique healthcare needs of MSM in relation to HIV prevention, treatment and care. Evidence from Kenya (Scheibe et al. 2017) suggests that acceptance of MSM as having health needs and equal rights in accessing healthcare was possible following sensitisation training on MSM-friendly services. In addition, findings from another study were that MSM avoided going to health facilities and chose self-care for STIs and other conditions as a result of negative attitudes by health providers (Kushwaha et al. 2017). On the other hand, a study by Fobosi et al. (2017) established that use of differentiated integrated service delivery approaches, such as use of night-time roadside wellness clinics promoted client satisfaction. This was largely because of the flexible operational hours as well as the friendliness of the services. Richter and Buthelezi (2021) in a systematic review conducted in Africa found that positive experiences of sex workers were noted amongst sex workers particularly when accessing healthcare services in the few sex worker-specific clinics.

### Stigma and discrimination

Stigma and discrimination towards FSWs is a commonly reported phenomenon in the literature. For example, Spyrelis and Ibisomi (2022) noted how sex workers complained about the judgemental attitudes of nurses when they came to healthcare facilities with symptoms of STIs. A study conducted in South Africa and Zambia by Krishnaratne et al. (2020) demonstrated highest levels of stigma and judgement by healthcare workers towards FSW and MSM. The same authors further highlighted the fact that stigma and discrimination towards key populations could have a bearing on the uptake of HIV care services and related treatment outcomes. Similar or related findings on stigma and discrimination as well as negative attitudes of healthcare providers were also highlighted in a South African study by Duby et al. (2019). In related studies in Zimbabwe, similar findings were noted, where stigma and discrimination in healthcare facilities were found to be a key barrier for key populations accessing HIV care services (Hunt et al. 2017; Moyo & Macherera 2021). A study in Kenya by Graham et al. (2018) found that trust in providers of care by the MSM was important in the adherence and enhancement of treatment continuity. The study further established that some MSM defaulted treatment or disengaged from care when their preferred provider of care was not available. In another, but related study, Shannon et al. (2018) attribute the hesitancy to seek HIV care services by sex workers to the previously experienced stigma and discrimination in healthcare settings.

## Discussion

This paper lays the groundwork for combating the challenges of a non-inclusive environment, negative attitudes of healthcare providers and stigma and discrimination encountered by key populations as they interface with the healthcare system by using Ubuntu. Admittedly, there is a paucity of literature on the application of Ubuntu concept in healthcare settings in Southern Africa, but our findings are in line with findings elsewhere exploring the values, ethos and principles of Ubuntu when combating HIV-related concerns in general. Some of the studies are by Mulaudzi (2012), Magezi and Khlopa (2021) and Tarkang, Pencille and Komesuor (2018).

### Non-inclusive healthcare environment

The narrative of a non-inclusive environment cuts across sub-Saharan Africa: Kushwaha et al. (2017), Ross et al. (2014), Larsson et al. (2016) and Wanyenze et al. (2016). Findings of a study by Nyato et al. (2019) revealed that the use of dedicated consultation rooms for HIV testing and ART initiation for key populations created a non-inclusive environment as there was a perception of stigma leading to hesitancy in accessing HIV care services. A related study by Ssekamatte et al. (2020) established that the healthcare environment was non-inclusive for transgender people. It was characterised by such barriers as inflexible hours of operation, social exclusion, breaches of confidentiality and stigmatisation. For that reason, it is important to heed the advice of Broodryk (2005) who advocates for the use of Ubuntu in the local language of patients in healthcare settings. The authors, therefore, argue that sensitisation trainings of healthcare providers attending to HIV-positive key population must use Ubuntu as understood in the local languages. According to Rasweswe et al. (2023), this approach seems to deliver positive healthcare outcomes in HIV healthcare settings. Another facet of Ubuntu in keeping with African epistemology is ‘I am because we are’, ‘Umuntu ngumuntu ngabantu’ (Mokhachane et al. 2023). They stress the importance of community which a healthcare setting should be. The Nguni also have an important saying ‘Akusilima sindebende kwabo’, meaning the family does not see the disability of any of its members. It would be wise and sensible for all healthcare providers dealing with HIV positive, to adopt that philosophy.

### Attitudes of healthcare workers

The negative attitudes of healthcare workers serve as barriers for key populations accessing HIV care services: Kigombola et al. (2023) and Kushwaha et al. (2017). Another, but related, South African study by Mulemfo et al. (2023) found that heterocentric healthcare service approaches tended to exclude LGBTQI+ people from gaining access to HIV care services. The aphorism ‘Umuntu ngumuntu ngabantu’ – a human being is a human being through others is a call to be non-judgemental in our interaction with others. Metz (2015) says that it is a call for altruism, consideration, compassion, empathy and solidarity. Therefore, a healthcare provider displaying all these attributes in his or her care for patients, regardless of their sexual orientation, is displaying Ubuntu. Ewuoso (2021) posits that Ubuntu rationalism is not indifferent to the needs of others and instead sees them as worthy of aid. The reviewed literature also demonstrated that some providers of care displayed positive attitudes (Kapanda et al. 2019; Kushwaha et al. 2017; Scheibe et al. 2017; Van der Elst et al. 2013). The Fobosi et al. (2017) study on roadside wellness clinics revealed participants’ satisfaction, particularly the night-time operational hours and the comprehensiveness of the services offered as well as the friendliness of the staff. Similarly, Richter and Buthelezi (2021) noted that recipients of care experienced friendly healthcare service provision particularly when these services had been tailor-made for key populations. These attitudes are in keeping with the nursing ethos advocated for by Henderson (1969) and resonate with the Ubuntu philosophy. In that respect, a sensitisation training encompassing Virginia Henderson ethos and underpinned by the Ubuntu values would see a challenge in attitudes by healthcare workers. A professional work ethic informed by Ubuntu would result in positive health outcomes for key populations receiving HIV prevention, treatment and care services.

### Stigma and discrimination

Evidence has demonstrated that key populations suffer stigma and discrimination when they access HIV care services (Graham et al. 2018; Hunt et al. 2017; Krishnaratne et al. 2020; Moyo & Macherera 2021). The hesitancy to seek HIV care services by sex workers is attributed to the previously experienced stigma and discrimination in healthcare settings according to a study by Shannon et al. (2018). Instances of stigma and discrimination were also noted in a Zambian study by Spyrelis and Ibisomi (2022). The sex workers reported how the nurses were rude to them, verbally abusive and blaming them for having acquired sexually transmitted infections (STIs). Rasweswe et al. (2023) argue that the application of Ubuntu norms and values ensured that those infected with HIV were treated with dignity as Ubuntu rules helped in destigmatisation. A study by Banda and Mudzanire (2019) emphasised the treatment of people living with HIV with dignity and respect. Key populations deserve similar treatment to the general population. This is concurred to by Ewuoso (2021), as Ubuntuism, unlike Judeo-Christian philosophies, proposes that the physical being of an individual entitles him to respect and dignity. Therefore, applying Ubuntu ethics in the provision of HIV care for key populations can prevent stigmatisation and homophobic tendencies. This will result in key populations being able to access HIV care services in a dignified manner as they experience the compassion and humanity embodied by Ubuntu. A study by Mokgatle and Madiba (2023) found that stigma and discriminatory attitudes were prevalent in South Africa. The authors are of the opinion that this could be worse with regard to HIV-positive key populations who would benefit from care provision informed by Ubuntu philosophy. In related studies, Gari et al. (2013) and Katz et al. (2013) demonstrated that stigma could act as a barrier to HIV testing and treatment uptake. Brandelli Costa et al. (2022) postulated that stigma and discrimination against key populations negatively impact their mental health, their uptake of HIV care services and adherence to treatment. This could be addressed if healthcare providers adopted the Ubuntu philosophy.

Olivier and Luies (2023) acknowledge the importance of social dynamics in the fight against HIV. Part of that acknowledgement should be that Ubuntu is a social contract that can be used to inform and destigmatise the healthcare environment for HIV-positive key populations. Thus, it is crucial to instil Ubuntu ideals and work ethics in healthcare providers involved in the provision of HIV care services.

### Limitations

This integrative literature review had the following limitations: it did not cover northern Africa. Therefore, its interpretation is limited to the regions covered. In addition, the review did not include grey literature but confined itself to peer-reviewed studies.

### Recommendations

The study recommends building capacity on KP-friendly services for communities, law enforcement and healthcare providers, further engagement of communities including religious leaders on key population issues and implementing differentiated service delivery models for key populations.

As demonstrated in a study by Dijkstra et al. (2021), peer networks informed by the Ubuntu philosophy of ‘I am because you are’ can be used to reach key population communities to facilitate the provision of HIV care services.

Both pre-service and in-service training for healthcare providers should instil Ubuntu philosophies in them, particularly around addressing the unique needs of key population sub-groups, a point further elaborated on by Mwango et al. (2022).

## Conclusion

This study brings insights into the challenges experienced by key populations as they access HIV care services. It also provides possibilities of how the challenges could be addressed using the Ubuntu philosophy. The lessons learnt in this study can be used by healthcare providers to provide KP-friendly services that are informed by Ubuntu philosophy. There is evidence (Duby et al. 2019; Scheibe et al. 2017) that focusing on sensitivities around HIV-care issues in various training programmes and adding it to key priority areas, has worked before. These could be further enriched by including in these programmes principles of the Ubuntu philosophy.
